# Thickness and Ratio of Noncompacted and Compacted Layers of the Left Ventricular Myocardium Evaluated in 56 Normal Fetuses by Two-Dimensional Echocardiography

**DOI:** 10.1155/2019/3726846

**Published:** 2019-01-23

**Authors:** Lei Sun, Enfa Zhao, Yajuan Wei, Chunmiao Kang, Baomin Liu

**Affiliations:** ^1^Department of Ultrasound, The Second Affiliated Hospital of Xi'an Jiaotong University, Xi'an, Shaanxi, China; ^2^Department of Ultrasound, Shaanxi Xi'an Electric Power Center Hospital, Xi'an, Shaanxi, China; ^3^Department of Structural Heart Disease, The First Affiliated Hospital of Xi'an Jiaotong University, Xi'an, Shaanxi, China

## Abstract

The thickness and ratio of noncompacted and compacted layers of the left ventricular (LV) myocardium in the normal fetus were investigated by fetal echocardiography. We aimed to investigate the compaction process of the LV myocardium during the normal gestation period and provide reference for echocardiographic diagnosis of a fetus with ventricular myocardium noncompaction. A total of 56 pregnant women in the gestational period of 23–30 weeks were included. Complete fetal echocardiography was performed with system ultrasonographic examination to exclude congenital heart malformation or extracardiac malformation. All 56 fetuses showed normal development. In the short-axis view of the fetal heart, the LV wall was divided into an upper and lower section at the level of the papillary muscle. Each section was then further divided into four segments, namely, anterior, posterior, lateral, and inferior wall. Thus, the LV wall was divided into eight segments. The thickness of the ventricular noncompacted and compacted layers and the ratio of the ventricular noncompacted to compacted layers of these segments at end-systole were measured and calculated. In echocardiography, the fetal LV myocardium is a two-layered structure: the endocardial noncompact myocardium (NC) with higher echo and the epicardium compact myocardium (C) with lower echo. The noncompacted layer is thinner than the compacted layer in the anterior wall, but thicker than the compacted layers in the posterior, lateral, and inferior wall. With respect to the upper and lower sections of the LV myocardium, the noncompacted layer in each segment of the upper section is thinner than that in each segment of the lower section, whereas the compacted layer of the upper section is thicker than that of the lower section. This study suggests that the densification of the fetal LV myocardium occurs gradually from base to apex and from the anterior to lateral, posterior, and inferior walls. This finding aids in further understanding the process of myocardial densification and provides a diagnostic reference for noncompaction of noncompaction cardiomyopathy (NCCM).

## 1. Introduction

Noncompaction cardiomyopathy (NCCM) is a rare cardiomyopathy that is characterized by excessively prominent trabeculae in the biventricular and deep intertrabecular recesses [1]. Trabecular compaction is usually more complete in the LV myocardium than in the right ventricular myocardium. Noncompaction of ventricular myocardium is believed to indicate an arrest in endomyocardial morphogenesis in the embryo [2]. The main pathological change is that the honeycomb-like reliefs form the inner layer of the ventricular structure, which divides the ventricular chamber into several small communicating ventricular cavities [3]. NCCM has three classical clinical presentations, namely, heart failure, arrhythmias, and embolic events [4, 5]. However, clinical presentation is highly variable and may range from incidental findings in asymptomatic patients to unexplained end-stage heart failure. The mortality rate associated with NCCM was up to 35% over a mean follow-up period of 3.7 years [6]. Thus, early diagnosis and treatment of patients with NCCM is crucial as high morbidity and mortality caused by life-threatening conditions [7]. Echocardiography is regarded as the first-choice diagnostic modality to determine myocardial pathologies [8]. There are three criteria for the diagnosis of NCCM [9–11], of which Jenni's criteria are the most commonly used. In Jenni's criteria, the ratio of the thickness of the noncompact layer to that of the compact layer is more than 2 [9–11]. However, to our knowledge, no studies have investigated the thickness and ratio of the noncompact and compact layers of fetuses in the normal myocardium, and there have been no relevant studies in fetuses. The aim of the present study was to illustrate the densification process of the LV myocardium in the normal fetus and provide reference for echocardiographic diagnosis of a fetus with ventricular myocardium noncompaction.

## 2. Methods

### 2.1. Study Subjects

We prospectively enrolled 56 pregnant women (mean age 27.52±2.74 years, gestational age 23-30 weeks) who were referred to the echocardiography department of our hospital for prenatal examination between February 2016 and April 2016. Routine and prenatal system examinations were performed to exclude potential cardiac abnormalities. All fetuses showed normal development. All patients had previously provided written informed consent. The study protocol was approved by the ethics committee of the Second Affiliated Hospital of Xi'an Jiaotong University.

### 2.2. Fetal Echocardiography

**Figure 1:**
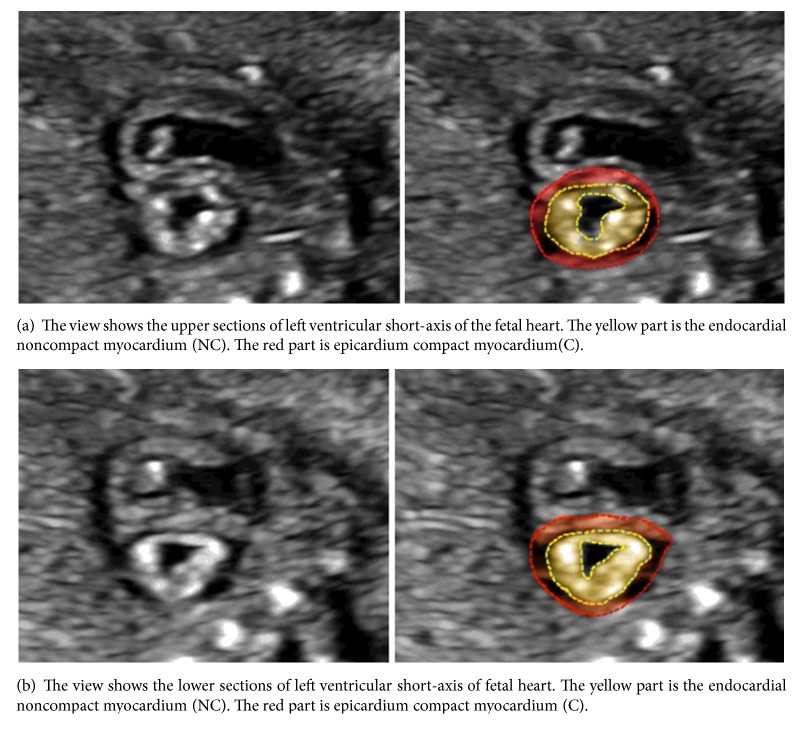


**Figure 2:**
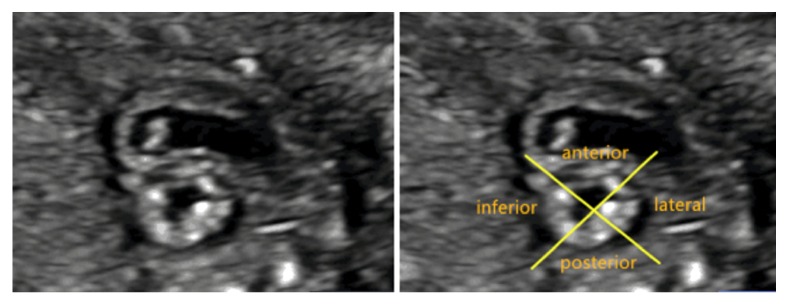
In the short-axis view of the heart, the LV wall was divided into four segments, namely, anterior, posterior, lateral, and inferior wall.

Fetal echocardiography was performed with Voluson E8 ultrasound scanner (GE Healthcare, Milwaukee, WI) using a RAB6-D transducer with 2–8 MHz (BT12). The examination was performed under the written consent of each patient, and pregnant women were kept in the supine or left lateral position, if needed. In the short-axis view of the heart, the LV wall was divided into an upper and lower section at the level of the papillary muscle (Figures 1(a) and 1(b)). Then, the two sections were further divided into four segments: anterior, posterior, lateral, and inferior wall (Figure 2). Thus, the LV wall was divided into eight segments. The thickness of ventricular NC and C of these segments at end-systole was measured, and NC/C ratio was calculated.

### 2.3. Statistical Analysis

Statistical analyses were performed using SPSS version 23.0 (SPSS, Chicago, IL). All measurement data were expressed as mean ± standard deviation (x±s). One-way ANOVA was performed to compare the NC/C ratio in the four divisions of the LV wall. The NC/C ratios in the upper and lower sections were compared using paired-sample t-test. A two-tailed P-value of < 0.05 was used as a cutoff for statistical significance.

## 3. Results

### 3.1. Echocardiography Features

**Figure 3:**
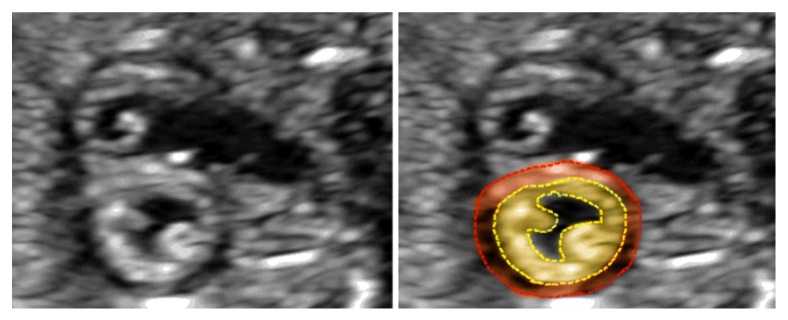
The view shows the level of the papillary muscle in the short-axis of fetal heart. The yellow part is the endocardial noncompact myocardium (NC). The red part is epicardium compact myocardium(C).

The muscular layer of the LV wall of the fetal heart can be divided into two layers according to the ultrasound image, that is, the compaction layer and noncompaction layer. The compaction layer is located in the epicardial myocardium, which is surrounded by a hypoechoic circular layer with a uniform internal structure. The noncompaction layer is located in the endocardial myocardium, which is hyperechoic and surrounds the inner side of the myocardium of the heart. Its internal structure is less compact, and the side near the endocardium is uneven with some small trabecular protuberances. This structure is more prominent from the bottom to the apex, and the most apparent two-layered structure boundary was present at the level of the papillary muscle (Figure 3).

### 3.2. Thickness and Ratio of NC and C Layers of the LV Myocardium in the Normal Fetus

The specific thickness of all 8 segments of the compacted and noncompacted layers was measured, and the NC/C ratios of the LV myocardium were calculated. All parameters mentioned above were measured in triplicate to obtain the mean value, and the NC/C ratios were determined (Table 1). The NC/C ratios between the upper and lower sections were compared. The average NC/C ratio was 1.034 (95% CI, 0.985–1.082) in the upper section, but 1.706 (95% CI, 1.619–1.794) in the lower section (Table 2). The NC/C ratio difference between the upper and lower sections was significant, as revealed by independent sample test (mean difference=-0.673; 95% CI, -0.77–-0.573; P<0.001). The NC/C ratio in the upper section was smaller than that in the lower section.

The NC/C ratios in the four segments were further compared. The NC/C ratios of the LV myocardium was 0.807 in the anterior wall (95% CI, 0.730–0.883), 1.460 in the posterior wall (95% CI, 1.383–1.537), 1.674 in the lateral wall (95% CI, 1.597–1.751), and 1.593 in the inferior wall (95% CI, 1.462–1.619) (Table 3(a)). One-way ANOVA revealed that the difference between N/C ratio between the anterior wall and the other three walls was significant (P<0.001). There was a significant difference in the NC/C ratio between the posterior and lateral walls as well as the lateral and inferior walls (P<0.05, respectively). However, there was no statistically significant difference in N/C ratio between the posterior and inferior walls (P=0.156) (Table 3(b)). There was a statistically significant difference in the NC/C ratio between the anterior wall and the other three walls. The anterior wall had a smaller NC/C ratio than the other three walls.

## 4. Discussion

NCCM is an increasingly recognized type of cardiomyopathy with the presence of an extensive trabeculated myocardium separated into two distinct layers: NC and C [12, 13]. In the early 4–6 weeks of embryonic development, the myocardium blood was supplied by the sinus space between muscle cells. If the heart develops defectively, development of the sinusoidal space between the myocardium fails, and the densification process of reticular muscle trabeculae also fails, resulting in the persistence of the trabecular myocardium and cardiac changes characterized by more prominent muscle trabeculae and deep trabecular recess. When the development of myocardium is blocked earlier, the range of the cardiac insufficiency becomes wider. Genetics play a vital role in NCCM development; about 17% to 50% of patients have a family member suffering from cardiomyopathy [6, 14]. It has been reported that the yield of DNA testing ranges from 17% to 41%, depending on the number of genes screened and patients selected [15, 16]. A recent study found that approximately one-half of the patients with NCCM had a mutation in a cardiomyopathy gene, and mutations in the* MYH7*,* MYBPC3*, and* TTN* genes were the most prevalent [17, 18]. Presently, in NCCM there is no radical treatment, and the prognosis of the disease is varied. If the lesion range is small, patients can be permanently asymptomatic. However, if the lesion range is wide, patients can develop progressive heart failure or ventricular arrhythmia at an earlier stage of life. Thus, early detection of NCCM can help to early intervention and treatment to delay the progression of heart failure and improve the quality of life.

Our study revealed that the myocardium layer of the LV of the fetal heart can be divided into two layers according to the ultrasound image, that is, the C myocardium and N myocardium. The two-layered structure of the myocardium shown on the ultrasound closely corresponds with the embryonic development of the heart [19]. At the fourth week of normal embryonic development, the myocardium is composed of a spongy reticular layer before the formation of the coronary artery circulation. The blood in the heart chamber supplies the corresponding region of the myocardium through the recess between them. At 5–8 weeks, the ventricular myocardium gradually becomes compact. The recess is compressed into capillaries, and the coronary microcirculation system gradually develops. The compaction process begins from the epicardium to endocardium, from the basis to the apex. As a result, muscle bundles are absorbed constantly, which results in the constant expansion of the ventricular cavity. By the 12th week of embryonic development, the compaction process is completed. The myocardium near the epicardium is completely formed, while the myocardium near the endocardium is not as completely formed as that near the epicardium. Consequently, some pectinate muscles and trabeculae remain. The smooth part of the interventricular septum is called the trabecular part because of the more complete absorption of the upper segment of the interventricular septum, while the lower segment of the ventricular septum is called the trabecularized part because of the incomplete absorption of the inferior segment of the ventricular septum. The other walls of the ventricles also have additional small muscle bundles due to inadequate absorption. If the compaction process is blocked due to some pathological factors, hypertrabeculation is formed [20]. When the development of myocardium is blocked at an early stage, the range of cardiac insufficiencies becomes wider. Therefore, the two-layered structure shown by echocardiography can reflect the formation and evolution of ventricular myocardium. Simultaneously, it was reported that NC/C ratio of the myocardium of normal children and adults is about 0.5–0.6 [21, 22]. However, in this study, the NC/C ratio of myocardium in the normal fetus was between 1 and 2, which was much higher than that of children and adults, suggesting that the compaction of fetal LV myocardium has not yet been completed, and the development of compaction of the fetal LV is a gradual development process. Our study revealed that compared with the NC/C ratio in the lower section of myocardium, the ratio in the upper section was smaller. The NC/C ratio of the anterior wall was smaller when compared to those in the posterior, lateral, and inferior walls. This suggested that the progressive process of myocardium compaction is from the upper section to the lower section and from the anterior wall to posterior, lateral, and inferior walls. This study showed that the degree of fetal myocardial compaction was very different from that of children and adults, and it provides a better understanding of the fetal myocardial compaction process as well as the pathological changes of hypertrabeculation.

However, our study also has limitations. This study is only a preliminary study of the NC and C layers of the fetal heart, and the study is limited to fetuses with a gestational age of 23–31 weeks. We could systematically study the fetal cardiac compaction and noncompaction layer and their ratios at different time points during early, middle, and late pregnancy in a future study. If such observations can be made in the future, it will aid in the elucidation of the complete process of human heart compaction during embryonic development. Furthermore, because the ratio of the NC/C layer depends on the location where it is measured, the results strongly depend on the observer. The reproducibility of the results and interobserver variability should not be ignored. Thus, a future study is needed to confirm this conclusion.

## Figures and Tables

**Table 1:** The thickness and ratio of left ventricular noncompact (NC) and compact (C).

Section	Segment	Number	NC (mm) Mean ± Std. Deviation	C (mm) Mean ±Std. Deviation	NC/C Mean ± Std. Deviation	95% Confidence Interval for Difference	
						Lower Bound	Upper Bound
Upper section	Anterior	56	1.423±0.361	2.546±0.582	0.577±0.164	0.534	0.620
	Posterior	56	1.800±0.430	1.575±0.260	1.160±0.300	1.080	1.240
	Lateral	56	2.016±0.405	1.675±0.309	1.237±0.294	1.158	1.316
	Inferior	56	1.927±0.381	1.693±0.296	1.161±0.245	1.096	1.227

Lower section	Anterior	56	1.968±0.410	2.220±0.837	1.036±0.590	0.878	1.194
	Posterior	56	2.030±0.385	1.205±0.286	1.761±0.483	1.631	1.890
	Lateral	56	2.352±0.517	1.148±0.246	2.112±0.553	1.964	2.260
	Inferior	56	2.009±0.362	1.088±0.219	1.917±0.472	1.790	2.043

**Table 2:** Ratio of NC/C of two sections of left ventricular myocardial in normal fetus.

	Number	Mean ± Std. Deviation	95% Confidence Interval for Difference	
			Lower Bound	Upper Bound
Upper section	224	1.034±0.368	0.985	1.082
Lower section	224	1.706±0.663	1.619	1.794

**Table:** (a) Ratio of NC/C of four segments of left ventricular myocardial in normal fetus

	Number	Mean ±Std. Deviation	95% Confidence Interval for Difference	
			Lower Bound	Upper Bound
Anterior	112	0.807±0.488	0.715	0.898
Posterior	112	1.460±0.501	1.367	1.554
Lateral	112	1.674±0.623	1.558	1.791
Inferior	112	1.539±0.533	1.439	1.639

**Table:** (b) Comparison of ratio of NC/C of four segments of left ventricular myocardial

(I) Divisions	(J) Divisions	Mean Difference (I-J)	Std. Error	Sig.	95% Confidence Interval for Difference	
					Lower Bound	Upper Bound
Anterior	Posterior	-0.654	0.055	<0.001^*∗*^	-0.763	-0.545
	Lateral	-0.868	0.055	<0.001^*∗*^	-0.977	-0.759
	Inferior	-0.732	0.055	<0.001^*∗*^	-0.841	-0.624

Posterior	Anterior	0.654	0.0551	<0.001^*∗*^	0.545	0.763
	Lateral	-0.214	0.055	<0.001^*∗*^	-0.323	-0.105
	Inferior	-0.079	0.055	0.156	-0.187	0.030

Lateral	Anterior	0.868	0.055	<0.001^*∗*^	0.759	0.977
	Posterior	0.214	0.055	<0.001^*∗*^	0.105	0.323
	Inferior	0.135	0.055	0.015^*∗*^	0.027	0.244

Inferior	Anterior	0.732	0.055	<0.001^*∗*^	0.624	0.841
	Posterior	0.079	0.055	0.156	-0.030	0.187
	Lateral	-0.135	0.055	0.015^*∗*^	-0.244	-0.027

^*∗*^The mean difference is significant at the 0.05 level.

## Data Availability

The data used to support the findings of this study are available from the corresponding author upon request.
